# Alkylphenol Activity against *Candida* spp. and *Microsporum canis*: A Focus on the Antifungal Activity of Thymol, Eugenol and *O*-Methyl Derivatives 

**DOI:** 10.3390/molecules16086422

**Published:** 2011-07-29

**Authors:** Raquel O.S. Fontenelle, Selene M. Morais, Erika H.S. Brito, Raimunda S.N. Brilhante, Rossana A. Cordeiro, Ynayara C. Lima, Nilce V.G.P.S. Brasil, André J. Monteiro, José J.C. Sidrim, Marcos F.G. Rocha

**Affiliations:** 1 Centre of the Agricultural Sciences and Biological, Acaraú Valley State University, 62040-370, Sobral, CE, Brazil; 2 Department of Chemistry, State University of Ceará, 60740-000, Fortaleza, CE, Brazil; Email: selene@uece.br; 3 Department of the Veterinary, Faculty of Veterinary Medicine, Superior Institute of Applied Theology, 62050-100, Sobral, CE, Brazil; Email: sallesbrito@yahoo.com.br; 4 Department of Pathology and Legal Medicine, Faculty of Medicine, Medical Mycology Specialized Center, Federal University of Ceará, 60441-750, Fortaleza, CE, Brazil; Email: brilhante@ufc.br (R.S.N.B.); ross_aguiar@yahoo.com.br (R.A.C.); sidrim@ufc.br (J.J.C.S.); mfgrocha@gmail.com (M.F.G.R.); 5 Department of Organic and Inorganic Chemistry, Federal University of Ceará, 60455-760, Fortaleza, CE, Brazil; Email: yna.colares@gmail.com (Y.C.L.) Nilcegramosapompeu@yahoo.com.br (N.V.G.); 6 Department of Statistics and Applied Mathematics, Federal University of Ceará, 60455-760, Fortaleza, CE, Brazil; Email: jalles@ufc.br; 7 Postgraduate Program in Veterinary Science, State University of Ceará, 60740-000, Fortaleza, CE, Brazil

**Keywords:** thymol, eugenol, estragole, methyl-derivatives, dermathophytes, *Candida*

## Abstract

In recent years there has been an increasing search for new antifungal compounds due to the side effects of conventional antifungal drugs and fungal resistance. The aims of this study were to test *in vitro* the activity of thymol, eugenol, estragole and anethole and some *O-*methyl-derivatives (methylthymol and methyleugenol) against *Candida* spp. and *Microsporum canis*. The broth microdilution method was used to determine the minimum inhibitory concentration (MIC). The minimum fungicidal concentrations (MFC) for both *Candida* spp. and *M. canis* were found by subculturing each fungal suspension on potato dextrose agar. Thymol, methylthymol, eugenol, methyl-eugenol, anethole, estragole and griseofulvin respectively, presented the following MIC values against *M. canis*: 4.8–9.7; 78–150; 39; 78–150; 78–150; 19–39 µg/mL and 0.006–2.5 μg/mL. The MFC values for all compounds ranged from 9.7 to 31 µg/mL. Concerning *Candida* spp*,* thymol, methylthymol, eugenol, methyleugenol, anethole, estragole and amphotericin, respectively, showed the following MIC values: 39; 620–1250; 150–620; 310–620; 620; 620–1250 and 0.25–2.0 μg/mL. The MFC values varied from 78 to 2500 µg/mL. All tested compounds thus showed *in vitro* antifungal activity against *Candida* spp. and *M. canis*. Therefore, further studies should be carried out to confirm the usefulness of these alkylphenols *in vivo*.

## 1. Introduction

Dermatophytosis is the most important superficial mycoses in both humans and animals. This clinical condition is caused by a group of related filamentous fungi of the genera *Epidermophyton*, *Microsporum* and *Trichophyton* [1]*.* The conventional treatment of fungal diseases is limited compared to antibiotic therapy for bacterial infections [2]. Treatment of dermatophyte infection involves primarily oral and/or topical formulations of azoles or allylamines, particularly itraconazole and terbinafine [3]. More recently, the increasing problems of antifungal drug resistance of dermatophytic fungi have been described [4]. 

The various forms of candidiasis are the most frequent causes of fungal infection in humans. A progressive increase in the frequency of candidemia has been observed, particularly among patients receiving antibiotics, immunosuppressive therapy or parenteral nutrition, as well as among patients exposed to invasive medical procedures [5]. Antifungal resistance in *Candida* spp strains has been observed in both humans and animals [5,6,7]. The resistance of *Candida* spp to currently available antifungal drugs represents a major challenge for future empirical therapeutic and prophylactic strategies. 

Therefore, numerous essential oils have been tested for both their *in vitro* and *in vivo* antimycotic activity. These oils have great potential as antifungal agents for use alone or together with standard antifungal therapies [2]. Essential oils that contain alkylphenols such as thymol, eugenol, estragole, and anethole have demonstrated antifungal properties [8,9,10,11,12].

**Figure 1:**
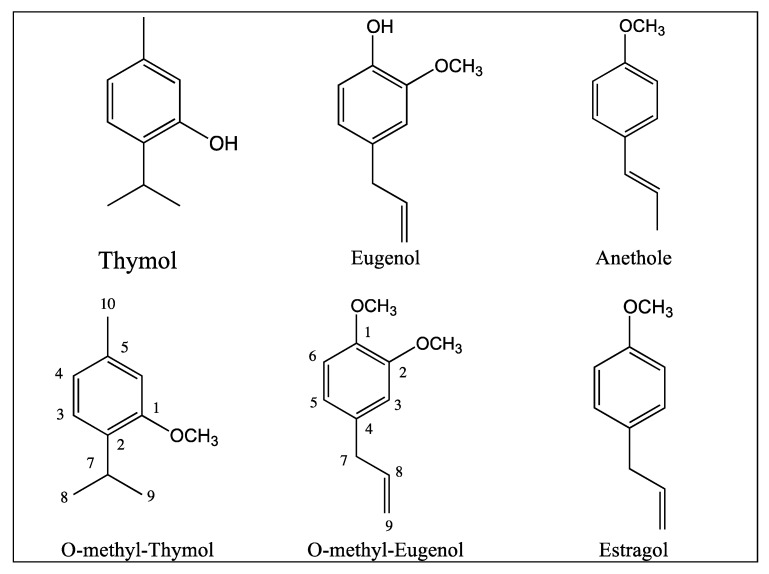
Chemical structures of antifungal alkylphenols.

Our previous studies with essential oils from species of *Crotons* [10] and *Lippia sidoides* [8] have shown their antifungal activity against *Candida* spp. and *M. canis*. In the GC/MS analysis of these oils, the main constituent found in the essential oil of *L. sidoides* was thymol, while in the essential oil of *Croton nepetaefolius* this was methyleugenol and for *Croton zenhtneri* its was estragole/anethole. Based on these studies, the present research was carried out to evaluate the antifungal activity of alkylphenols (thymol, eugenol, estragole and anethole) and *O-*methyl-derivatives of thymol and eugenol (Figure 1) against *Candida* spp. and *M. canis in vitro*. The *O-*methyl-derivatives were obtained by reaction of the phenolic group with dimethyl sulfate in alkaline medium [13].

## 2. Results and Discussion

The screening results for the antifungal activity of the alkylphenols (thymol, eugenol, estragole and anethole) and thymol/eugenol methyl-derivatives against *M. canis* and *Candida* spp. are shown in Table 1, while Table 2 shows the MICs and MFCs of thymol, methylthymol, eugenol, methyleugenol, anethol and estragole against *M. canis*. A statistically significant difference was observed when comparing MICs (p = 0.0256) and MFCs (p = 0.0256) for thymol to MICs (p = 0.0231) and MFCs (p = 0.0231) for eugenol against *M*. *canis* strains.

Table 2 also shows the MICs and MFCs of thymol, methylthymol, eugenol, methyleugenol, anethole and estragole against *Candida* spp. There was statistical significance for the MIC (p = 0.0196) and MFC (p = 0.0196) of thymol in relation to the other alkylphenols.

Many essential oils exhibit antioxidant and antimicrobial activities. Phenols, such as thymol, carvacrol, and eugenol, are among the most active natural antioxidants and antimicrobials found in essential oils. However, due to their poor water solubility and the requirement for high concentrations to reach a therapeutic effect, the efficiency of these compounds in treatment is limited [14].

**Table 1:** Screening of antifungal activity of alkylphenols against *M. canis* and *Candida* spp. by the agar-well diffusion method.

	**Growth Inhibition Zones (mm)**							
	**Alkylphenols** (10,000 µg/mL)						**Controls**	
**Strains**	thymol	methyl-thymol	eugenol	methyl-eugenol	anethole	estragole	griseofulvin (1000 µg/mL)	amphotericin B (5 µg/mL)
***M. canis***								
CEMM 01-3-188	35	22	26	20	16	23	55	-
CEMM 01-5-190	40	20	30	20	20	20	47	-
***C.albicans***								
CEMM 01-3-075	18	18	12	10	11	11	-	14
CEMM 01-3-069	17	13	8	7	9	8	-	10

Essential oils rich in alkylphenols frequently present antimicrobial activity [2]. This has been corroborated by several other studies on the effect of essential oils of *Croton* species and *Lippia sidoides*, which are rich in thymol, methyl-eugenol, estragole and anethole, and were shown to have antifungal properties against *Candida* spp. and *M. canis* [8,10]. In addition, the antimicrobial activity of thymol was demonstrated against oral pathogens [9]; the inhibitory effects of anethole and eugenol were evaluated based on the growth and toxin production of *Aspergillus parasiticus* [14].

Eugenol was shown to be effective against fungi isolated from onychomycosis [15]; a fungistatic dodecanol combined with a sublethal amount of anethole showed fungicidal activity against *S. cerevisiae* [16] and the anti-*Candida* effects of estragole in combination with ketoconazole or amphotericin B was demonstrated [17].

This work demonstrates the great potential of alkylphenols as antifungal agents against *Candida* spp. and *Microsporum canis*, as observed by the relevant MIC and MFC levels of thymol, anethole, estragole and methyl-derivatives (methylthymol and methyleugenol). Although all the tested compounds showed MIC and MFC values, which support their antimicrobial activity against fungi of medical interest, thymol stood out for its potent effect *in* *vitro* on both the dermatophyte *M.* *canis* and the *Candida* spp.

Corroborating these results, Botelho *et al.* [9] demonstrated that thymol has potent antimicrobial activity against *Candida albicans*, with minimum inhibitory concentrations ranging from 625 to 10,000 µg/mL. More recently, Guo *et al.* [18] showed the antifungal activity of thymol against clinical isolates of fluconazole-sensitive and -resistant *Candida albicans* strains. In addition, Gayoso *et al.* [15] showed that eugenol is a strong antifungal agent against yeast (*C. albicans*, *C. tropicalis*, *C. krusei*) and dermathophytes (*T. rubrum* and *T. mentagrophytes*), suggesting this alkylphenol is a promising compound for the development of antifungal drugs. It was also reported that anethole shows synergistic effects on the antifungal activities of phytochemicals against *Saccharomyces cerevisiae* and *C. albicans* [16]. 

**Table 2:** Minimum inhibitory and fungicidal concentrations of alkylphenols against *M. Canis* and *Candida* spp. evaluated by the broth microdilution method.

	**Alkylphenols (µg/mL)**												**Controls (** **μg/mL)**	
**Strains**	thymol		methyl-thymol		eugenol		methyl-eugenol		anethole		estragole		amphotericin B	griseofulvin
	MIC	MFC	MIC	MFC	MIC	MFC	MIC	MFC	MIC	MFC	MIC	MFC	MIC	MIC
*C. albicans* (n = 4)	39 (4)a	78 (4)	1250 (4)	2500 (4)	620 (3)	1250 (3)	620 (3)	1250 (3)	620 (4)	1250 (4)	1250 (3)	2500 (3)	0.5 (2)	-
					150 (1)	310 (1)	310 (1)	620 (1)			620 (1)	1250 (1)	0.25 (2)	
*C. tropicalis* (n = 2)	39 (2)	78 (2)	1250 (1)	2500 (1)	310 (2)	620 (2)	310 (2)	620 (2)	620 (2)	1250 (2)	620 (2)	1250 (2)	1.0 (1)	-
			620 (1)	1250 (1)									0.5 (1)	
*C. krusei* (n = 1)	150	310	1250	2500	620	1250	620	1250	620	1250	620	1250	0.5	-
ATCC 22019														
*C. parapsiloses* (n = 1)	150	310	1250	2500	620	1250	620	1250	620	1250	620	1250	2.0	-
ATCC 6528														
*M. canis* (n = 6)	9.7 (3)	19 (3)	150 (4)	310 (4)	39 (6)	78 (6)	150 (2)	310 (2)	150 (3)	310 (3)	39 (4)	78 (4)	-	0.25 (2)
	4.8 (3)	9.7 (3)	78 (2)	150 (2)			78 (4)	150 (4)	78 (3)	150 (3)	19 (2)	39 (2)		0.125 (3)
														0.006 (1)

^a^ Represents the number of strains of a species for the MIC and MFC indicated.

## 3. Experimental

### 3.1. General

Estragole, anethole and eugenol were purchased from Sigma Chemical Co. (St. Louis, MO, USA) and thymol was supplied by Vetec (Duque de Caxias, RJ, Brazil). The methyl-derivatives were synthesized as previously described by Furniss *et al.* [13]. Briefly, eugenol or thymol (0.05 mol) was suspended in a cold solution of NaOH (13.3 g, 0.33 mol) in water (81.25 mL) with vigorous stirring. Dimethyl sulfate (14.4 g, 0.12 mol) was added in one portion, and the mixture was shaken vigorously for 20 minutes, while the temperature was maintained below 35 °C by external cooling. A second portion of dimethyl sulfate (14.4 g, 0.12 mol) was added and the temperature was allowed to rise to 40–45 °C. At the end of the reaction, the reaction mixture was transferred to a separatory funnel. The organic layer, containing the methyl-derivatives, was washed with distilled water and dried with anhydrous sodium sulfate. The solvent was then evaporated to furnish the corresponding methyl derivatives. The chloroform was eliminated in a rotary evaporator to obtain pure methyl derivatives, which were analyzed by ^1^H-, ^13^C-NMR and mass spectroscopy [19]. The presence of the absorption band in 55.2 δ of the OCH_3_ group in the ^13^C-NMR and the corresponding peak at 3.87 δ in the ^1^H-NMR spectrum of *O-*methylthymol confirmed the methylation of thymol. In the spectra of *O-*methyl-eugenol, the mass spectra confirmed the molecular mass at *m/z* 178, in the ^1^H-NMR spectrum two peaks at 3.88 δ and 3.90 δ appeared, while in the ^13^C-NMR there were also two peaks for the methoxyl groups at 55.7 δ and 55.9 δ. The spectral data of the methyl derivatives of thymol and eugenol are listed below. 

*O-Methylthymol.* ^13^C-NMR (δ, CDCl_3_): 155.6 (C1), 136.3 (C2), 126.8 (C3), 121.1 (C4), 134 (C5), 111.4 (C6), 25.4 (C7), 22.8 (C8), 22.8 (C9), 21.3 (C10), 55.2 (OCH_3_). ^1^H-NMR (δ, CDCl_3_): 1.26 (d, 6.4, CH_3_8/CH_3_9), 2.39 (s, CH_3_7), 3.34 (h, 6.4, H7), 3.87 (s, OCH_3_), 6.73 (s, H6), 6.81 (d, 5.6, H3), 7.17 (d, 5.6, H4).

*O-Methyleugenol.* MS-EI *m/z* (Int. %): 178 (100), 163 (45), 151 (15), 147 (50), 131 (14), 115 (26), 103 (78), 91 (84), 77 (39), 65 (38), 51 (22). ^13^C-NMR (δ, CDCl_3_): 147.3 (C1), 148.8 (C2), 111.2 (C3), 132.6 (C4), 120.4 (C5), 111.8 (C6), 39.7 (C7), 137.8 (C8), 115.6 (C9), 95.9 (C10), 95.7 (C11). ^1^H-NMR (δ, CDCl_3_): 3.36 (d, 2.5, H7), 3.88 (OCH_3_), 3.90 (OCH_3_), 5.09 (dd, 9.3; 2.9, H9), 5.98 (m, H8), 6.76 (d, 4.7, H-5), 6.83 (m, H3, H6).

### 3.2. Fungal strains

The strains were obtained from the fungus collection of the Specialized Medical Mycology Center—CEMM (Federal University of Ceará, Brazil), where they were maintained in saline (0.9% NaCl), at 28 °C. At the time of the analysis, an aliquot of each suspension was taken and inoculated on potato dextrose agar (DIFCO, Detroit, MI, USA), and then incubated at 28 °C for 2–10 days. A total of six strains of *M. canis* and six *Candida* spp. (four *C. albicans* and two *C. tropicalis*) were included in this study. Both *M. canis* and *Candida* spp. strains were isolated from dogs. In addition, *C. parapsilosis* (ATCC 22 019) and *C. krusei* (ATCC 6528) strains were used as a quality controls. 

### 3.3. Agar-well diffusion method

The inocula preparation for the agar-well diffusion method was carried out as previously described [8,10,20]. The screening of the antifungal activity of the alkylphenols and methyl derivatives of thymol and eugenol was performed against *Candida* spp and *M. canis* strains by the agar-well diffusion method, as previous reported [8,10,20]. In brief, Petri dishes (15 cm in diameter) were prepared with potato dextrose agar (DIFCO) and then wells (6 mm in diameter) were cut from the agar. Each fungal suspension was inoculated on the surface of the agar and 0.100 mL of each suspension was placed into the wells. After incubation at 28 °C, for 3–5 days for *Candida* spp. and 5–8 days for *M. canis*, all the dishes were examined for growth-inhibition zones and the diameters of these zones were measured in millimeters. Each experiment was repeated at least twice. The alkylphenols and methyl-derivatives were dissolved in DMSO to obtain a test concentration of10,000 µg/mL. griseofulvin (1000 µg/mL; Sigma Chemical Co.) and amphotericin B (5 µg/mL; Sigma Chemical Co.) were prepared in distilled water and tested as positive controls for *M. canis* and *Candida* spp., respectively.

### 3.4. Broth microdilution method

For the broth microdilution method, the inocula preparation was carried out as previously described [6,7,8,10]. The minimum inhibitory concentration (MIC) for *Candida* spp. was determined by the broth microdilution method, in accordance with the Clinical and Laboratory Standards Institute—CLSI (formerly NCCLS; M27-A2) [21]. The broth microdilution assay for *M. canis* was performed as described by Jessup *et al.* [22], Fernandez-Torres *et al.* [23]*,* and Brilhante *et al.* [24], based on the M38-A document [25]. The minimum fungicidal concentrations (MFC) for both *Candida* spp. and *M. canis* were found according Fontenelle *et al.* [8]. 

The microdilution assay was performed in 96-well microdilution plates and the alkylphenols were tested in concentrations ranging from 4 to 5,000 µg/mL. Growth and sterile control wells were included in each experiment. The microplates were incubated at 37 °C and read visually after two days for *Candida* spp. and five days for *M. canis*, as previously reported [6,7,10,20,23,25]. All isolates were run in duplicate. The MIC was defined as the lowest alkylphenol concentration that caused 100% inhibition of visible fungal growth. The results were read visually as recommended by CLSI. The MFC was determined by subculturing 100 µL of solution from subsequent MIC well on potato dextrose agar at 28 °C. The MFCs were determined as the lowest concentration resulting in no growth on the subculture after two days for *Candida* spp. and five days for *M. canis* [6,7,8,10].

### 3.5. Statistical analysis

The MICs and MFCs of the aromatic compounds and methyl derivatives were analyzed by the Wilcoxon and Friedman tests. The result was considered significant at p < 0.05. The followings comparisons were carried out: thymol *vs*. methyl-thymol; eugenol *vs*. methyl-eugenol; and differences among the isolated alkylphenols.

## 4. Conclusions

Our results demonstrate that alkylphenols showed *in vitro* antifungal activity against *Candida* spp. and *M. canis*. Since thymol and eugenol are skin irritants, further studies should be carried out to establish whether or not methyl derivatives of thymol and eugenol possess better pharmacokinetics and safety characteristics than their parent compounds for *in vivo* usefulness.
